# Monosodium glutamate‐mediated Ca^2+^‐dependent intestinal epithelial ion transports in health and IBS‐D in male mice

**DOI:** 10.14814/phy2.70975

**Published:** 2026-06-12

**Authors:** Yunxian Wang, Fenglan Chu, Yanhua Gong, Ruihong Guo, Hui Dong

**Affiliations:** ^1^ Department of Pharmacology School of Pharmacy, Qingdao University Medical College Qingdao China

**Keywords:** IBS‐D, IECs, intestinal ion transports, MSG, NCX

## Abstract

Despite the ubiquitous use of monosodium glutamate (MSG) as a food additive, its specific effects on intestinal health and disease remain largely unclear. Here we found mucosal application of MSG induced an upward intestinal short‐circuit current (*I*_*sc*_) in the murine ileum, which was significantly reduced by extracellular Cl^−^, Na^+^, and Ca^2+^ removal. Mechanistically, MSG‐induced *I*_*sc*_ was significantly attenuated by pharmacological inhibition of key transporters (NCX, SGLT1, NKA) and secretory channels (CaCC, CFTR, IK_Ca_). Furthermore, disrupting intracellular Ca^2+^ homeostasis via endoplasmic reticulum (ER) chelation or blockade of IP_3_R, RyR, SOCE/CRAC, and VGCC significantly suppressed both the ileal *I*_*sc*_ and MSG‐stimulated Ca^2+^ signaling in IECs. Importantly, MSG triggered a simultaneous elevation in intracellular Ca^2+^ and a reduction in Na^+^, which were abolished by the blockers of NCX and SGLT1. In vivo, MSG‐induced Ca^2+^‐dependent ion transports were significantly reduced in irritable bowel syndrome with diarrhea (IBS‐D) mice. Our findings indicate that MSG drives Ca^2+^‐dependent intestinal ion transports through a complex network orchestrated by SGLT1 and NCX under healthy state, which is markedly attenuated in IBS‐D. This study provides novel insights into the gastrointestinal impact of dietary MSG in both health and bowel disease.

## 1 INTRODUCTION

Monosodium glutamate (MSG) is one of the most widely used human food additives in the world and is often added to soup bases and meat products (Bellisle, 1999; Zanfirescu et al., 2019). In the past three decades, the consumption of MSG has increased significantly worldwide (Yachmin et al., 2021), and China has also taken a leading position in global consumption, production, and export of MSG to other countries (Kayode et al., 2023). After intake into the gastrointestinal (GI) tract, MSG is decomposed by digestive enzymes, generating different products, including glutamic acid and sodium ions (Na^+^). Most of the glutamic acid (up to 95%) in the diet is used as an energy source for intestinal epithelial cells (IECs); others are rapidly metabolized by IECs and liver cells (Burrin & Stoll, 2009), and supplied for use by various cells in the body (Williams & Woessner, 2009). Na^+^ is absorbed into the body to maintain fluid balance and to exert many other biological activities as well.

The intake of MSG induces several physiological processes, such as taste stimulation (Diepeveen et al., 2022), participation in protein metabolism, energy metabolism, nitrogen metabolism (Tomankova et al., 2015), and influence on sodium intake (Hien et al., 2024), etc. In addition, MSG may also exert a variety of pharmacological effects. Some evidence shows that MSG has different impacts on the development of obesity, type 2 diabetes (Bahadoran et al., 2019), hypertension, and metabolic syndrome (Chakraborty, 2019). While the physiological and pharmacological effects of MSG have been extensively investigated outside the GI tract (Singh & Panda, n.d.; Kasmara et al., 2025; Vorhees, 2018), surprisingly, the effects of MSG on the GI tract and IECs are largely unclear although the GI tract is the first organ for MSG intake to enter the body.

Intestinal epithelial ion transport is an important physiological process in the GI tract, in which Ca^2+^ plays a critical regulatory role (Kiela & Ghishan, 2009). By regulating a variety of ion channels and transporters, Ca^2+^ affects epithelial ion absorption and secretion by the intestine (Cui et al., 2021; Murek et al., 2010). It is generally believed that some secretagogues (such as acetylcholine) mainly conduct Ca^2+^ signaling through the classical Ca^2+^ pool or store‐operated calcium entry (SOCE) mechanism in IECs. This mechanism mainly includes two important processes: the release of Ca^2+^ from intracellular stores and the entry of extracellular Ca^2+^ into cells (West et al., 2022). IP_3_‐sensitive and RyR‐sensitive Ca^2+^ stores have been identified in the endoplasmic reticulum (ER). The former is activated by the binding of IP_3_ and the IP_3_ receptor (IP_3_R), and the latter is activated by the binding of ryanodine and the ryanodine receptor (RyR) to induce the ER Ca^2+^ release into the cytoplasmic solution (Lin et al., 2016; Yue et al., 2020). In addition, previous studies also showed that the Ca^2+^ release‐activated Ca^2+^ channel (CRAC) is a highly selective Ca^2+^ store‐operated Ca^2+^ channel, which may have a certain correlation with SOCE (Kodakandla et al., 2022). The IP_3_R/RyR‐mediated Ca^2+^ release constitutes a key component of the SOCE or CRAC/Orai channel (Cui et al., 2021). However, it is currently unclear whether Ca^2+^ is pivotal for the regulation of MSG ingestion and whether the SOCE mechanism is involved in MSG‐mediated epithelial ion transport.

NCX is a sodium/calcium transporter on the cell membrane and plays a crucial role in numerous physiological processes. Extensive research has been conducted on NCX in the brain (Omelchenko et al., 2019), heart (Szlovák et al., 2021), and other systems, yet its role in GI health and diseases remains largely unknown. As a bidirectional transporter on the cell membrane, NCX exists in two transport modes: in the Ca^2+^ efflux mode, 3 Na^+^ enter the cell while 1 Ca^2+^ is extruded; in the Ca^2+^ influx mode, 3 Na^+^ are extruded from the cell accompanied by the entry of 1 Ca^2+^ (Giladi et al., 2016). Moreover, it is acknowledged that SGLT1 plays a critical role in intestinal Na^+^‐glucose uptake with a ratio of 2 Na^+^ to 1 glucose (Zhang et al., 2021). However, at present, it remains unclear whether NCX and SGLT1 play roles in MSG‐mediated Ca^2+^‐dependent ion transport and, if so, which transport mode of NCX is involved in this process.

Irritable bowel syndrome (IBS), a common functional GI disease, is characterized by abdominal pain, bloating, changes in bowel habits, and stool shape (Enck et al., 2016). IBS is considered a chronic gut‐brain disorder with a global incidence rate as high as 9%–20% since the disordered communication between the GI tract and the brain leads to motor disorders, visceral hypersensitivity, and changes in central nervous system processing (Huang et al., 2023; Sebastián Domingo, 2022). As the most commonly used food additive, MSG has well‐known central neural effects (Kim et al., 2025; Ureña‐Guerrero et al., 2003), and it has been identified as a triggering factor for abdominal pain in IBS (Brant et al., 2023), suggesting MSG ingestion is associated with the pathogenesis of IBS.

Therefore, in the present study, we investigated if MSG as a world‐widely used food additive affects intestinal epithelial ion transport, and if so, what are the underlying cellular mechanisms. Afterwards, we investigated whether MSG‐mediated epithelial ion transports are involved in the pathogenesis of IBS. We demonstrate for the first time that MSG mediates Ca^2+^‐dependent intestinal ion transport via activation of multiple epithelial ion channels and transporters, particularly NCX in IECs, implying the possible involvements of MSG in GI health and disease. MSG‐induced intestinal ion transports are likely changed during IBS‐D.

## 2 METHODS

### 2.1 Animal study

All animal experiments carried out comply with ARRIVE guidelines 2.0 for reporting animal research (Percie du Sert et al., 2020). The animal use protocol has been reviewed and approved by the Ethics Committee of the Medical College of Qingdao University (QDU‐AEC‐2025102) and was conducted in compliance with its guidelines. All animal care and experimental procedures complied with the NIH Guide for the Care and Use of Laboratory Animals, and animal conduct conformed to the ARRIVE guidelines (Percie du Sert et al., 2020). All experiments used adult male C57BL/6 mice, which were 6–8 weeks old and weighed 18–22 grams (these mice were purchased from Jinan Pengyue Experimental Animal Breeding Co., Ltd., China; mouse food was provided by Shandong Pengyue Laboratory Animal Technology Co., Ltd., PY‐025). The mice were housed in polypropylene plastic cages. They were kept in a room with an ambient temperature controlled at 20°C–30°C and a humidity of 50%–55%, and were exposed to a 12/24‐h light–dark cycle. They had free access to water and food. Before the experiments, the mice were anesthetized with 100% carbon dioxide (CO_2_) and sacrificed with cervical dislocation. In all experiments, animals were randomly selected and pooled. Data collection and evaluation were performed blindly, and the experimenters were unaware of the group treatments.

### 2.2 Cell culture

IEC‐6 (RRID: CVCL_0343), a small intestinal epithelial cell line of rat origin (Thomas & Oates, 2002), was purchased from the Chinese Academy of Sciences (Shanghai, China). All cells were cultured in DMEM‐high glucose medium (HyClone, USA) supplemented with 10% fetal bovine serum (HyClone, USA), 1% penicillin/streptomycin (Invitrogen, USA). All cells were grown in a 37°C humidified atmosphere containing 5% CO_2_. After the cells had grown well for experiments, they were replated onto 12 mm round coverslips (Warner Instruments Inc., Hamden, CT, United States) and incubated for at least 24 h before use for cytosolic Ca^2+^ concentration ([Ca^2+^]_i_) measurement.

### 2.3 Tissue preparation

After euthanizing the mice, the abdomen of each mouse was incised along the midline. In this research project, the mucosal tissues of the duodenum, jejunum, and ileum were mainly studied, with each tissue segment being approximately 4 cm in length. To ensure the viability of the intestinal epithelial tissues and avoid further damage to the intestinal epithelium, the isolated intestinal segments were incubated in a solution containing 10 mL of Krebs buffer and indomethacin. This was aimed at inhibiting the endogenous PGE_2_ that might be generated due to mucosal damage during the experiment, thus preventing any influence on the basal *I*_*sc*_. Krebs buffer (mM) contained 118 NaCl, 4.7 KCl, 1.18 MgSO_4_, 25 NaHCO_3_, 1.2 KH_2_PO_4_, 1.6 CaCl_2_, and 11.1 D‐glucose. Subsequently, the intestine was longitudinally incised along the mesentery, and the seromuscular layer was peeled off. Finally, the tissue was divided into 3–4 pieces of tissue with the size of a chamber (the window area was 0.1 cm^2^).

### 2.4 Ussing chamber experiments

The Ussing chamber experiment was conducted as previously described (Zhang et al., 2019). The processed intestinal segment tissues were fixed in a modified Ussing chamber with an exposed area of 0.1 cm^2^. The experiment was carried out under continuous short‐circuit conditions (voltage–current clamping, VCC MC6; Physiological Instruments, San Diego, California). The transepithelial short‐circuit current (*I*_*sc*_) was measured by an automatic voltage clamp. The original records were in μA, and the pooled data were in μA·cm^−2^. After measuring the basic parameters for 10–15 min, different agonists or inhibitors were added to the mucosal side, the serosal side, or both sides of the tissue for 10–20 min, and then MSG was added. The buffers on both sides of the Ussing chamber were prepared separately. The composition of the mucosal solution (mM) was 115 NaCl, 1.2 MgCl_2_, 1.2 CaCl_2_, 25 sodium gluconate, 5.2 potassium gluconate, and 10 D‐mannitol (with a final pH of 7.4), and it was aerated with 100% oxygen. The composition of the serosal solution (mM) was 115 NaCl, 1.2 MgCl_2_, 1.2 CaCl_2_, 25 NaHCO_3_, 2.2 K_2_HPO_4_, 0.8 KH_2_PO_4_, and 10 D‐glucose (with a final pH of 7.4), and it was in a carbogen mixture (5% CO_2_ and 95% O_2_, v/v). During the experiment, 3 mL of each of the above solutions was added to both sides of the chamber at a constant temperature of 37°C. To create a low Na^+^ environment on the mucosal side or the serosal side, the Na^+^ in the mucosal solution or the serosal solution was replaced with Li^+^ at the same concentration. Therefore, the composition of the mucosal low Na^+^ solution (mM) was 115 LiCl, 1.2 MgCl_2_, 1.2 CaCl_2_, 25 sodium gluconate, 5.2 potassium gluconate, and 10 D‐mannitol. The composition of the serosal low Na^+^ solution (mM) was 115 LiCl, 1.2 MgCl_2_, 1.2 CaCl_2_, 25 NaHCO_3_, 2.2 K_2_HPO_4_, 0.8 KH_2_PO_4_, and 10 D‐glucose. For the 0 Ca^2+^ experiment, the CaCl_2_ in the corresponding mucosal or serosal working solution was replaced with NaCl at the same concentration, and then EGTA was added to a final concentration of 0.5 mM to prevent Ca^2+^ contamination in the environment. When preparing the 0 Cl^−^ solution, NaCl, MgCl_2_, and CaCl_2_ were replaced with sodium gluconate, calcium gluconate, and magnesium gluconate.

### 2.5 Ca^2+^ imaging in IECs

The fluorescence Ca^2+^ imaging experiment was carried out as previously described (Wan et al., 2017). The cells cultured on the coverslips were incubated for 1 h at 37°C with a dye composed of 5 μM Fura‐2/AM (Solarbio, China, Cat. No. F1221) and physiological saline solution (PSS), and then washed with PSS for 20 min. Pluronic F‐127 (20% in DMSO) (Solarbio, China, Cat. No. F8460) was used in the dye preparation process to improve the solubilization of Fura‐2/AM and ensure its uniform intracellular distribution. When conducting experiments with different inhibitors or agonists, the inhibitors or agonists were incubated in the PSS solution for 20 min. Subsequently, the coverslips with cells were placed in the perfusion chamber of a fluorescence microscope (OLYMPUS IX73, Japan). The changes in Ca^2+^ signaling were monitored by tracking the excitation ratio of Fura‐2/AM at 340 nm and 380 nm. In our study, the fluorescence ratio of single cell was recorded every 3 s, so the sampling rate used in our study was 1/3 fs (Hz). The fluorescence signaling was imaged using an intensified charge‐coupled device (ICCD) camera (HAMAMATSU ORCA‐Flash4.0 LT, Japan) connected to an inverted fluorescence microscope (OLYMPUS IX73, Japan), and recorded with MetaFluor software (Molecular Devices Corporation, USA). The PSS used for digital Ca^2+^ measurement contained the following components (mM): 140 Na^+^, 5 K^+^, 2 Ca^2+^, 147 Cl^−^, 10 Hepes, and 10 glucose (pH 7.4).

### 2.6 Intracellular Na^+^ determination

Intracellular Na^+^ levels were determined as previously reported using Sodium Green™ (Cat. No. S6901, Invitrogen, USA) (Zhou et al., n.d.). Sodium Green™ was excited at 488 nm, and the fluorescence intensity under 488 nm excitation was recorded by F/F0.

### 2.7 IBS‐D animal model establishment

Wrap restraint stress (WRS) and water avoidance stress (WAS) have been introduced into scientific literature as models for human irritable bowel syndrome (IBS) (Xia et al., 2023; Xing et al., 2025). After 1 week of adaptive feeding, mice were randomly divided into 2 groups: a control group and a WRS model group. To minimize the influence of circadian rhythms, acute restraint stress was performed consecutively for 7 days between 9:00 am and 11:00 am. On the seventh day, fecal samples were collected from each mouse group and analyzed for weight measurement as well as moisture content determination along with quantification of distinguishable particles. For WAS model, mice were also randomly divided into a control group and a WAS model group. Acute water avoidance stress was applied for 3 h between 9:00 am and 12:00 am, after which fecal samples were collected from each group for weight measurement as well as moisture content determination along with quantification of distinguishable particles.

### 2.8 Materials

All salts and nifedipine were from Shanghai Aladdin Biochemical Technology Co., Sigma (Saint Louis, MO, United States) supplied monosodium glutamate (MSG), cyclopiazonic acid (CPA), and 4‐chloro‐3‐ethylphenol (4‐CEP). L‐glutamic acid, N, N, N′, N‐tetrakis (2‐pyridylmethyl) ethylenediamine (TPEN), dantrolene, xestospongin C, ouabain, SN‐6, 2‐aminoethyldiphenyl borinate (2‐APB), SKF‐96365, YM‐58483, NFA, and CFTR_inh_‐172 were purchased from MedChemExpress (MCE; Monmouth Junction, NJ, USA). LiCl, TRAM34, SN‐6, SEA0400, phlorizin, and canagliflozin were from Macklin (Shanghai, China). KCl and glucose were from Sangon Biotech (Shanghai, China). Detailed information on the reagents was presented in Table S1.

### 2.9 Data analysis

All experiments and data collection were performed blindly. All results are given as the mean ± SD. The net *I*_*sc*_ peak is calculated by subtracting the basal level from the maximum peak stimulated by the drug. Unpaired two‐tailed *t*‐tests or one‐way analysis of variance (ANOVA) followed by Dunnett's post‐hoc test were used to determine the statistical significance of the differences in means among the experimental groups. Only a probability of *p* < 0.05 was considered statistically significant. In animal experiments, *n* represents the numbers of experimental tissues. In Ca^2+^ imaging experiment, *n* represents the numbers of IEC‐6 cells.

## 3 RESULTS

### 3.1 MSG induces intestinal epithelial ion transport

**FIGURE 1:**
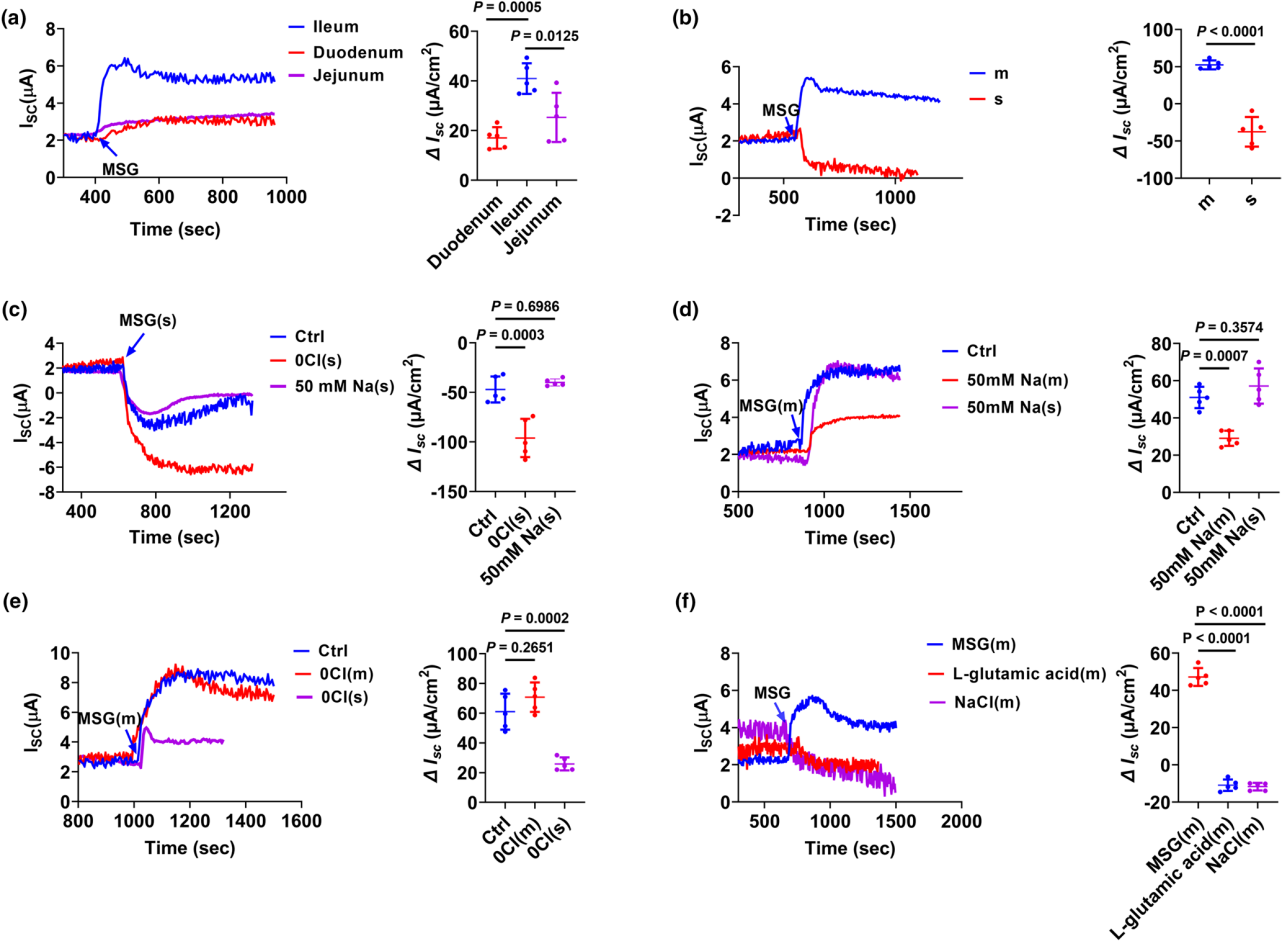
MSG induces intestinal epithelial ion transport. (a) Representative time courses and summary data of mucosal addition (indicated by the blue arrow) of MSG (25 mM)‐stimulated *I*_*sc*_ of the duodenum, proximal jejunum, and distal ileum (*n* = 5). (b) Representative time courses of MSG (25 mM)‐stimulated *I*_*sc*_ and the summary data of *I*_*sc*_ peak when added to the serosal (s) or mucosal (m) side of the ileum (*n* = 5). (c) Representative time courses and summary data of MSG‐stimulated *I*_*sc*_ peak on the serosal side after removing Cl^−^ from the serosal side [0Cl(s), *n* = 5] and removing Na^+^ from the serosal side [50 mM Na (s), Li^+^ replaced Na^+^, *n* = 5]. (d) Comparison of the distal ileum *I*_*sc*_ induced by mucosal side addition of MSG (25 mM) under normal Na^+^, mucosal low‐Na^+^ [50 mM Na (m), Li^+^ replaced Na^+^, *n* = 5], and serosal low‐Na^+^ conditions [50 mM Na (s), Li^+^ replaced Na^+^, *n* = 5]. (e) Representative time courses and summary data of MSG‐stimulated *I*_*sc*_ peak after Cl^−^ omission from the mucosal side [0Cl(m), *n* = 5] and the serosal side [0Cl(s), *n* = 5]. (f) Representative time courses and summary data of the *I*_*sc*_ induced by adding MSG (25 mM), L‐glutamic acid (25 mM), and NaCl (25 mM) respectively to the mucosal side of the distal ileum (*n* = 5).

Effects of MSG on the intestine are largely unclear although the intestine is the first organ for its entrance into the body. Therefore, we examined the effect of MSG on intestinal ion transport by Ussing chamber experiment to determine intestinal short‐circuit current (*I*_*sc*_) in mice. Firstly, we found that adding MSG (25 mM) into intestinal lumen induced an obvious *I*_*sc*_, with an instantaneous peak followed by a continuous phase. Secondly, we observed the segmental differences in MSG‐induced intestinal *I*_*sc*_. As shown in Figure 1a, MSG‐induced *I*_*sc*_ in the distal ileum was greater and faster than that induced in the duodenum and jejunum. Therefore, we used the distal ileum in the following Ussing chamber experiments.

Since the intestinal epithelium has obvious polarity (Massey‐Harroche, 2000), we explored which side addition of MSG induced ileal *I*_*sc*_. As shown in Figure 1b, mucosal application of MSG induced an upward ileal *I*_*sc*_, but serosal application induced a downward *I*_*sc*_. To examine the roles of Cl^−^ and Na^+^, we omitted Cl^−^ from the serosal side, which significantly enhanced MSG‐induced ileal *I*_*sc*_ on the serosal side, but reducing serosal Na^+^ showed no significant difference (Figure 1c). Therefore, Cl^−^ is likely involved in MSG‐induced serosal downward *I*_*sc*_.

Since MSG is absorbed from the intestinal mucosal side, we focused on its effects on this side. MSG‐induced upward ileal *I*_*sc*_ due to mucosal application suggests its stimulatory actions on Cl^−^ secretion. We also found that reducing Na^+^ from the mucosal side significantly reduced MSG‐induced ileal *I*_*sc*_, while there was no difference on the serosal side (Figure 1d). Meanwhile, omitting Cl^−^ from the mucosal side showed no significant difference, while it significantly attenuated MSG‐induced ileal *I*_*sc*_ on the serosal side (Figure 1e). In addition, considering that MSG can dissociate into L‐glutamic acid and Na^+^, we investigated whether the MSG‐induced upward ileal *I*_*sc*_ is attributable to MSG itself or to its individual components—L‐glutamic acid or Na^+^. The result demonstrated that the application of 25 mM L‐glutamic acid or 25 mM NaCl to the mucosal side each elicited a small downward *I*_*sc*_ (Figure 1f). These findings indicate that the MSG‐induced upward *I*_*sc*_ is not due to any single component but rather results from the synergistic effect of glutamic acid and Na^+^, a MSG‐induced specific ileal *I*_*sc*_.

### 3.2 CaCC and CFTR in MSG‐induced ileal Cl^−^ secretion

**FIGURE 2:**
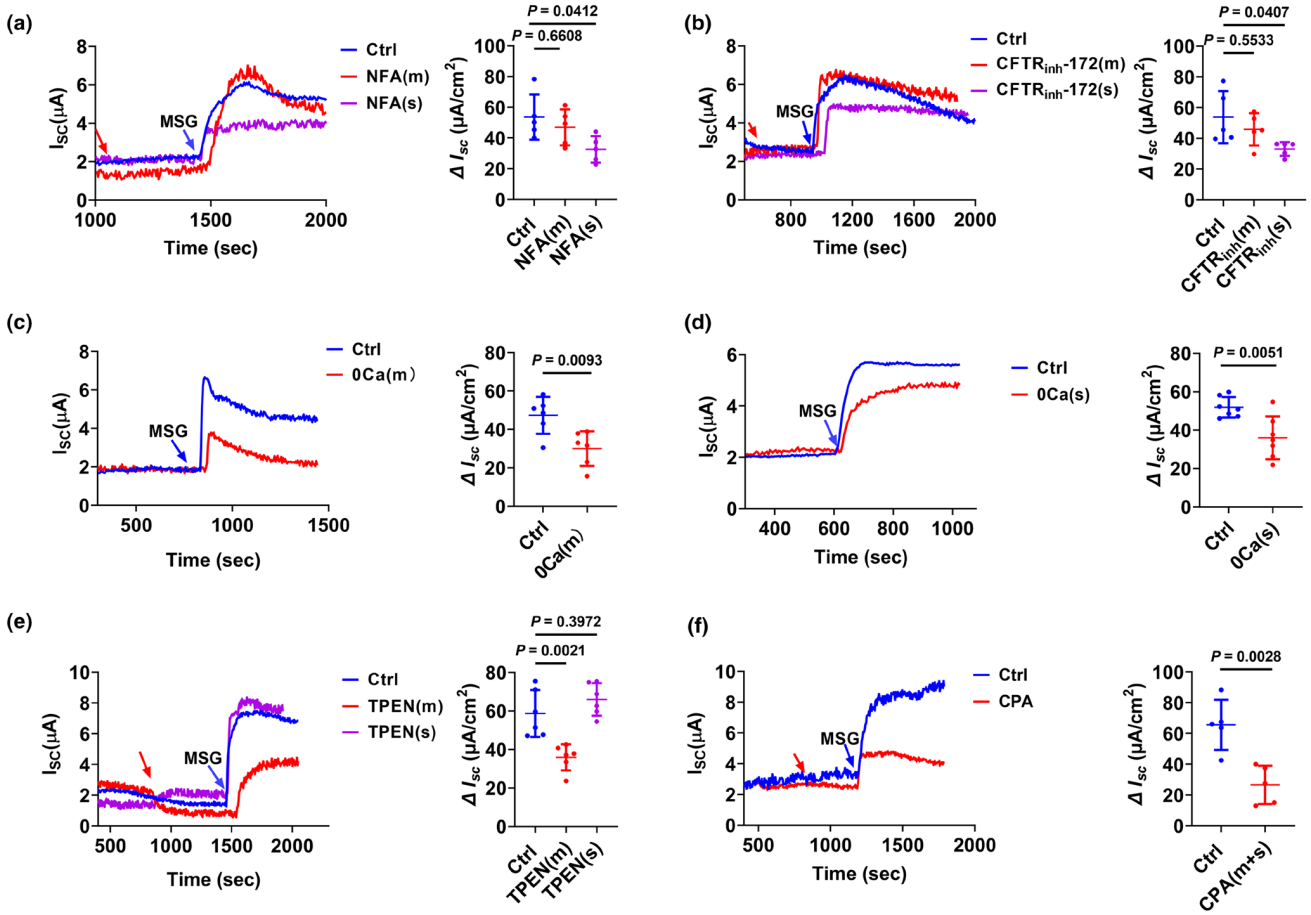
Roles of CaCC and CFTR in MSG‐induced ileal *I*_*sc*_, and extracellular/ER Ca^2+^ in MSG‐induced epithelial ion transport. (a) Representative time courses and summary data of MSG‐stimulated *I*_*sc*_ when NFA (200 μM, *n* = 5) was added to the mucosal side (m) or the serosal side (s). (b) Representative time courses and summary data of MSG‐stimulated *I*_*sc*_ when CFTR_inh_‐172 (30 μM, *n* = 5) was added to the mucosal side (m) or the serosal side (s). (c, d) Representative time courses and summary data of MSG (MSG, 25 mM)‐stimulated *I*_*sc*_ after the absence of extracellular Ca^2+^ (0 Ca) on the mucosal side (m) (*n* = 6) or the serosal side (s) (*n* = 6) of the ileal mucosal tissue. (e) Representative time courses and pooled data of MSG‐stimulated ileal *I*_*sc*_ after mucosal (m) addition of TPEN (1 mM, *n* = 6) or serosal (s) addition of TPEN (1 mM, *n* = 6). (f) Representative time courses and summary data of MSG‐stimulated ileal *I*_*sc*_ after adding CPA (30 μM, *n* = 5) on both sides (m + s) of the tissue.

We initially focused on MSG‐stimulated ileal Cl^−^ secretion. It is well known that CaCC and CFTR are crucial in intestinal epithelial Cl^−^ secretion stimulated by Ca^2+^‐mobilizing secretagogues (Harvey & McElvaney, 2024). Therefore, we applied CaCC blocker niflumic acid (NFA) (200 μM) (Zhang et al., 2019). Addition of it to the mucosal side had no effect on MSG‐induced ileal *I*_*sc*_, but it reduced MSG‐induced *I*_*sc*_ on the serosal side (Figure 2a). Similarly, CFTR inhibitor CFTR_inh_‐172 (30 μM) (Zhang et al., 2019) showed no effect when added to the mucosal side, but attenuated MSG‐induced ileal *I*_*sc*_ when administered on the serosal side (Figure 2b). Therefore, MSG induced intestinal Cl^−^ secretion through activation of both CaCC and CFTR.

### 3.3 Extracellular Ca^2+^ and ER Ca^2+^ storage in MSG‐induced epithelial ion transport

Ca^2+^ signaling in IECs plays a pivotal role in the regulation of intestinal epithelial ion transport (Chappell et al., 2008; Xie et al., 2014a). To test whether Ca^2+^ is crucial for MSG‐stimulated ileal *I*_*sc*_, we removed Ca^2+^ from either mucosal or serosal side, which significantly attenuated MSG‐induced *I*_*sc*_ (Figure 2c,d). Therefore, extracellular Ca^2+^ is essential for the full expression of MSG‐induced ileal *I*_*sc*_. We further investigated the role of ER Ca^2+^ storage in MSG‐induced ileal *I*_*sc*_. As shown in Figure 2e, when the ER Ca^2+^ chelator, N, N, N′, N′‐tetrakis (2‐pyridylmethyl) ethylenediamine (TPEN, 1 mM) (Chu et al., 2022) was applied to mucosal side, MSG‐induced ileal *I*_*sc*_ was significantly attenuated, but it was not affected after application to serosal side, suggesting that the ER Ca^2+^ storage that is close to mucosal side of the tissue plays a dominant role. Similarly, mucosal and serosal application of cyclopiazonic acid (CPA, 30 μM) (Chu et al., 2022) also significantly reduced MSG‐induced *I*_*sc*_ (Figure 2f). Therefore, MSG‐induced intestinal ion transport depends on both extracellular Ca^2+^ and the ER Ca^2+^ storage.

### 3.4 Intracellular Ca^2+^ release via IP_3_R and RyR in MSG‐induced ileal *I*_*sc*_

**FIGURE 3:**
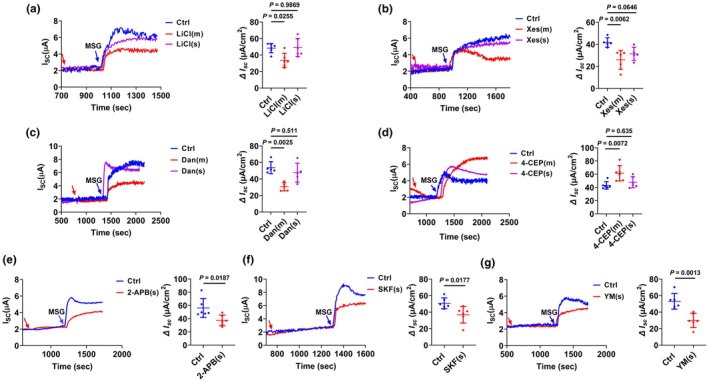
Intracellular Ca^2+^ release via IP_3_R/RyR and the SOCE/CRAC/Orai channels in MSG‐induced ileal *I*_*sc*_. (a) Representative time courses and summary data showing the effect of LiCl (30 mM, *n* = 6) on MSG‐stimulated *I*_*sc*_ after mucosal (m) addition or serosal (s) addition. (b) Representative time courses and summary data showing the effect of xestospongin C (Xes, 1 μM, *n* = 5) on MSG‐stimulated *I*_*sc*_ after mucosal (m) addition or serosal (s) addition. (c) Representative time courses and summary data showing the effect of dantrolene (Dan, 300 μM, *n* = 5) on MSG‐stimulated *I*_*sc*_ after mucosal (m) addition or serosal (s) addition. (d) Representative time courses and summary data showing the effect of 4‐CEP (1 mM, *n* = 6) on MSG‐stimulated *I*_*sc*_ after mucosal (m) addition or serosal (s) addition. (e) Representative time courses and summary data of MSG‐induced *I*_*sc*_ after serosal (s) application of 2‐aminoethoxydiphenyl borate (2‐APB, 100 μM, *n* = 6). (f) Representative time courses and summary data of MSG‐induced *I*_*sc*_ after serosal (s) application of SKF‐96365 (SKF, 30 μM, *n* = 6). (g) Representative time courses and summary data of MSG‐induced *I*_*sc*_ after serosal (s) application of YM‐58483 (YM, 0.3 μM, *n* = 6).

We further investigated whether two ER Ca^2+^ release pathways, IP_3_R and RyR, are involved in MSG‐induced ileal *I*_*sc*_. First, we used LiCl to inhibit the production of IP_3_. Mucosal application of LiCl (30 mM) (43) significantly weakened MSG‐induced *I*_*sc*_, but serosal application did not (Figure 3a). Similarly, mucosal application of xestospongin C (Xes, 1 μM), a selective IP_3_R inhibitor, significantly weakened MSG‐induced *I*_*sc*_, but serosal application did not (Figure 3b). Second, we used the selective RyR inhibitor dantrolene. Mucosal application of dantrolene (Dan, 300 μM) (Zhang et al., 2019) significantly inhibited MSG‐induced *I*_*sc*_, but serosal application did not (Figure 3c). Finally, the selective RyR agonist 4‐CEP was also used. Mucosal application of 4‐CEP (1 mM) significantly promoted MSG‐induced *I*_*sc*_, but serosal application did not (Figure 3d). Taken together, these data strongly suggest that intracellular Ca^2+^ release via IP_3_R and RyR from the ER that is close to the mucosal side of the intestine plays a dominant role in regulating MSG‐induced ileal *I*_*sc*_.

### 3.5 The SOCE/CRAC/Orai channels in MSG‐induced ileal *I*_*sc*_

The ER Ca^2+^ release may trigger the store‐operated calcium entry (SOCE) mechanism, an important physiological process within many types of cells, including IECs (Cui et al., 2021; Nguyen et al., 2018). To examine whether this mechanism is also involved in MSG‐induced intestinal ion transport, we used several selective SOCE blockers (Cui et al., 2021). Since the SOCE mechanism is exclusively present on the serosal membrane side (Chu et al., 2022; Cui et al., 2021; Kodakandla et al., 2023), we added its inhibitor only to the serosal side to observe its effect on MSG‐induced ileal *I*_*sc*_. As shown in Figure 3e, addition of 2‐APB (100 μM) (Chu et al., 2022) to the serosal side significantly reduced ileal *I*_*sc*_. Although 2‐APB is a reliable SOCE blocker, it is also an IP_3_R inhibitor (Gárriz et al., 2021). Therefore, SKF‐96365 (30 μM) (Zhang et al., 2019), another SOCE blocker with higher selectivity, was applied to the serosal side to significantly reduce ileal *I*_*sc*_ (Figure 3f). Since it is acknowledged that CRAC/Orai channels are the molecular candidate for the SOCE, we applied selective CRAC/Orai blocker YM‐58483 (0.3 μM) (Chu et al., 2022) to the serosal side, which significantly reduced MSG‐induced ileal *I*_*sc*_ (Figure 3g). Therefore, our data suggest that MSG induces ileal *I*_*sc*_ likely via activation of CRAC/Orai channels on the serosal side of the intestinal epithelium.

### 3.6 NCX and VGCC in MSG‐induced ileal *I*_*sc*_

**FIGURE 4:**
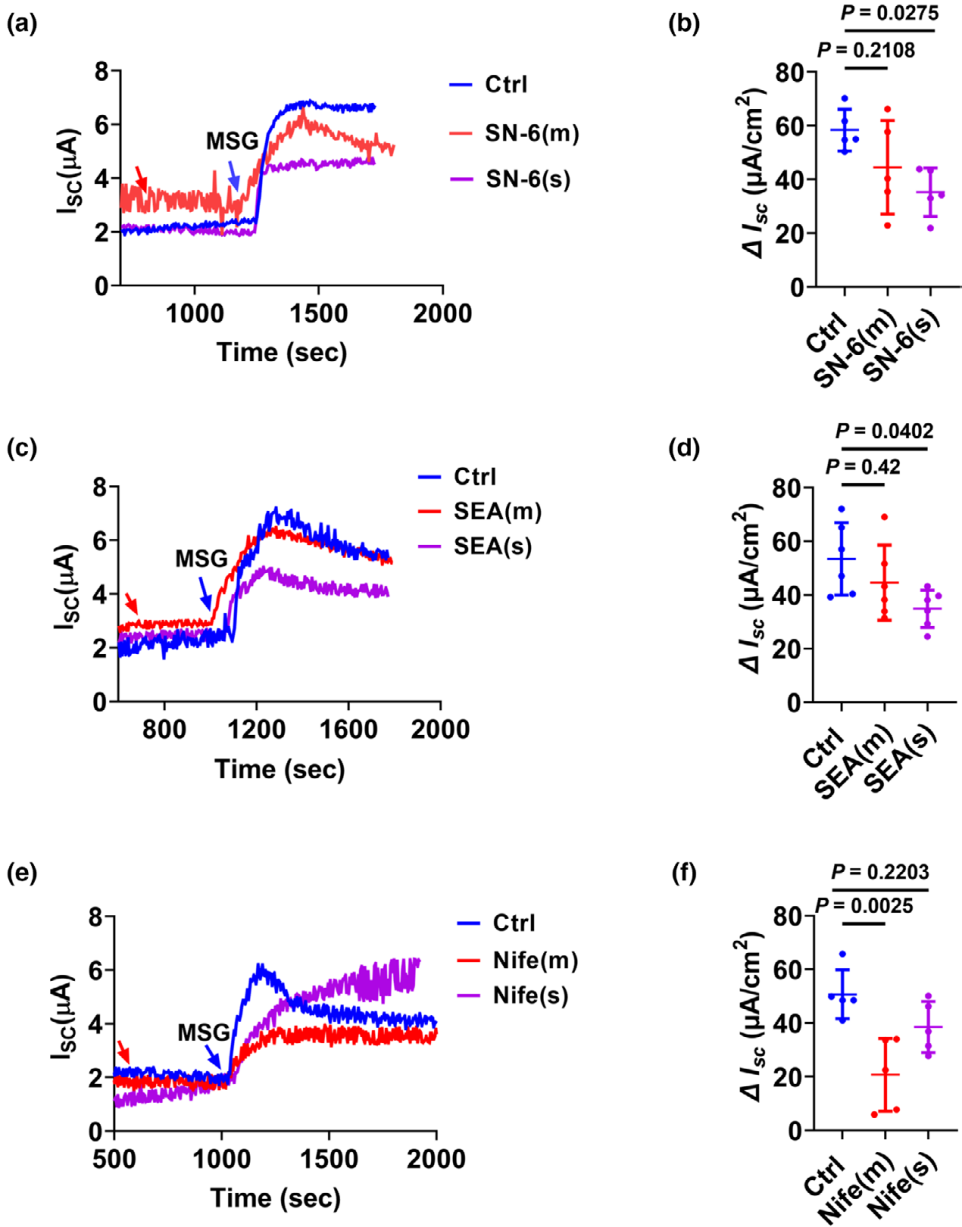
NCX and VGCC in MSG‐induced ileal *I*_*sc*_. (a, b) Representative time courses and summary data showing the effect of mucosal (m) or serosal (s) addition of SN‐6 (10 μM, *n* = 5) on MSG‐induced *I*_*sc*_. (c, d) Representative time courses and summary data showing the effect of mucosal (m) or serosal (s) addition of SEA0400 (SEA, 5 μM, *n* = 6) on MSG‐induced *I*_*sc*_. (e, f) Representative time courses and summary data showing the effect of mucosal (m) or serosal (s) addition of nifedipine (Nife, 10 μM, *n* = 5) on MSG‐induced *I*_*sc*_. All instances of “SEA” in the figure refer to SEA0400.

As a bidirectional transporter, NCX plays an important role in maintaining Ca^2+^ homeostasis of cell signaling (Liao et al., 2012). We firstly explored the role of NCX in MSG‐induced ileal *I*_*sc*_. Mucosal addition of selective NCX inhibitor SN‐6 (Kita & Iwamoto, 2007) (10 μM) (Chu et al., 2022) had no effect, but serosal addition significantly attenuated MSG‐induced *I*_*sc*_ (Figure 4a,b). Similarly, we applied another NCX inhibitor with higher selectivity, SEA0400 (Kuwahara et al., 2025). Mucosal addition had no significant effect, but serosal addition attenuated MSG‐induced *I*_*sc*_ (Figure 4c,d). Since the L‐VGCC are also functionally expressed in IECs (Beggs et al., 2019; Reyes‐Fernandez & Fleet, 2015), we secondly examined whether they are involved. While VGCC blocker nifedipine (10 μM) (Chu et al., 2022) was added to the mucosal side, it significantly attenuated MSG‐induced *I*_*sc*_, but serosal addition had no effect (Figure 4e,f). The inhibition percentage of the inhibitor on MSG‐induced ileal *I*_*sc*_ is shown in Table S2. Therefore, MSG induces ileal *I*_*sc*_ also via activation of NCX and VGCC in intestinal epithelium.

### 3.7 Na^+^/K^+^ ATPase (NKA) and IK_Ca_ in MSG‐induced ileal *I*_*sc*_

**FIGURE 5:**
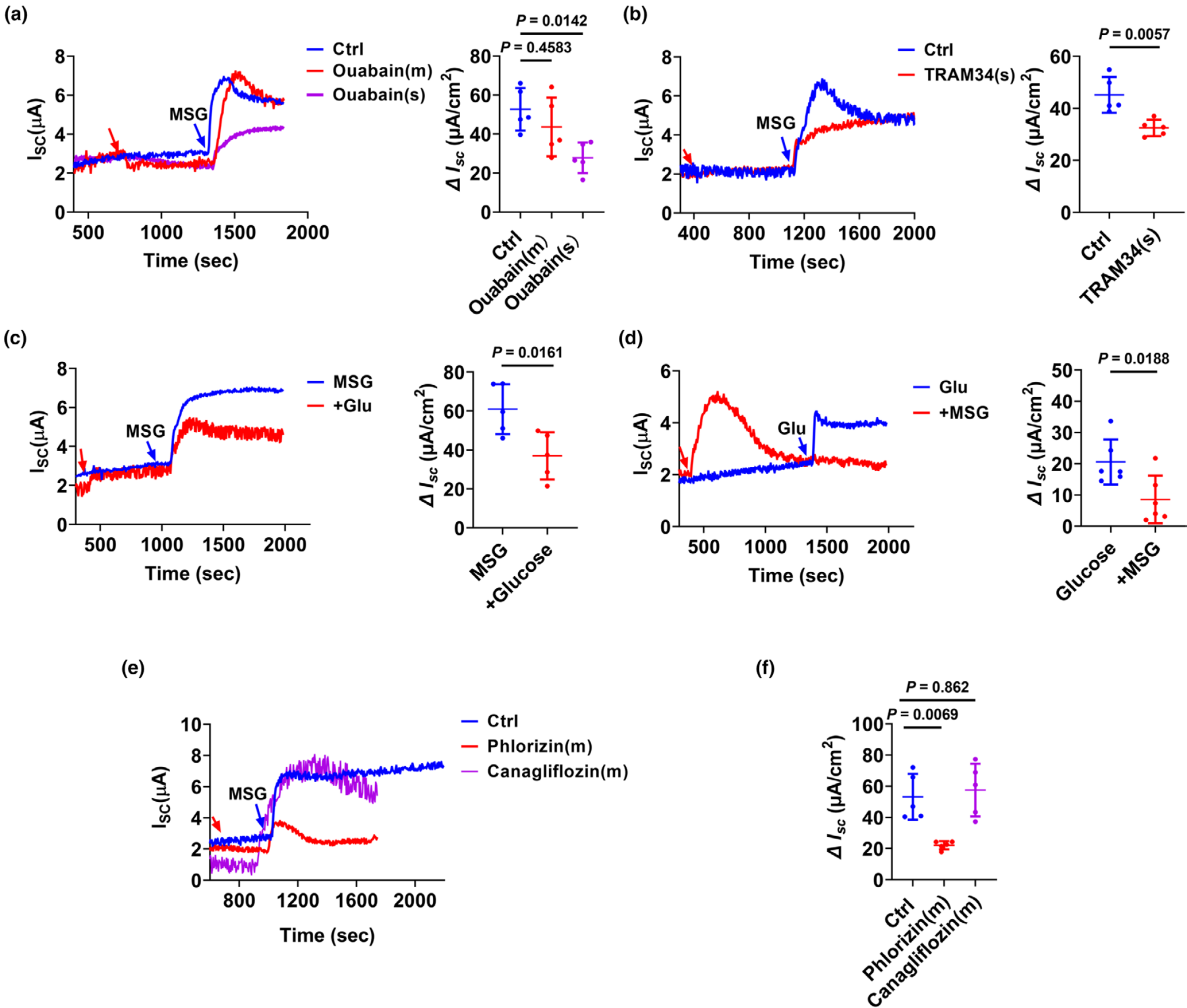
The roles of NKA, IK_Ca_, and SGLT1 in MSG‐induced ileal *I*_*sc*_. (a) Representative time courses and summary data showing the effect of mucosal (m) or serosal (s) addition of ouabain (1 mM, *n* = 5) on MSG‐induced *I*_*sc*_. (b) Representative time courses and summary data showing the effect of serosal (s) addition of TRAM34 (10 μM, *n* = 5) on MSG‐induced *I*_*sc*_. (c) Representative time courses and summary data of MSG (25 mM)‐stimulated *I*_*sc*_ after adding glucose (3 mM) to the mucosal side (m) (*n* = 5). (d) Representative time courses and summary data of glucose (3 mM)‐stimulated *I*_*sc*_ after adding MSG (10 mM) to the mucosal side (m) (*n* = 6). (e, f) Representative time courses and summary data of MSG‐stimulated *I*_*sc*_ when phlorizin (300 μM, *n* = 5) and canagliflozin (10 μM, *n* = 5) were added to the mucosal side (m).

NKA is critical for intestinal ion transport and involved in Ca^2+^ signaling transduction by coordinating the ion concentration gradient across the cell membrane (Huang et al., 2024). Moreover, IK_Ca_ works in concert with NKA to regulate the transmembrane gradient of Na^+^ (Félétou, 2016), and also to maintain the Na^+^ gradient and electrochemical equilibrium required by SGLT1 operation (Palaniappan et al., 2019). Therefore, we first investigated whether they are involved in MSG‐induced ileal *I*_*sc*_. Addition of the selective NKA inhibitor ouabain (1 mM) (Chu et al., 2022) to the mucosal side had no significant effect, but serosal addition significantly attenuated MSG‐induced ileal *I*_*sc*_ (Figure 5a). Similarly, since TRAM34 targets IK_Ca_ located on the serosal membrane side (Joiner et al., 2003; Xie et al., 2014; Xu et al., 2020), serosal addition of the selective IK_Ca_ blocker TRAM34 (Rehman et al., 2020) (10 μM) (Loganathan et al., 2011) significantly attenuated MSG‐induced ileal *I*_*sc*_ (Figure 5b). The inhibition percentage of the inhibitor on MSG‐induced ileal *I*_*sc*_ is shown in Table S2. In conclusion, both NKA and IK_Ca_ channels on the serosal side are crucial for MSG‐induced ileal *I*_*sc*_.

### 3.8 SGLT1 in MSG‐induced ileal *I*_*sc*_

It is acknowledged that intestinal glucose uptake is predominantly via SGLT1 and glucose induces intestinal *I*_*sc*_ by stimulating both Na^+^ absorption and Cl^−^ secretion (Zhang et al., 2021). Although MSG‐induced ileal *I*_*sc*_ was reduced by a low mucosal Na^+^ level (Figure 1d), it is unclear whether SGLT1 plays a role in MSG‐induced ileal *I*_*sc*_. To this end, firstly, glucose (3 mM) was added to the mucosal side of the ileum. When glucose‐induced *I*_*sc*_ reached the maximum (about 10 min later), mucosal application of MSG (25 mM) was applied. Compared to the control group, MSG‐induced ileal *I*_*sc*_ was significantly attenuated (Figure 5c). Vice versa, MSG (10 mM) pretreatment abolished 3 mM glucose‐induced *I*_*sc*_ (Figure 5d), suggesting that MSG and glucose may compete with each other for the same absorptive transporters.

Secondly, since SGLT1 is the main intestinal transporter for glucose absorption, we further verified whether it is also critical for MSG absorption. Indeed, since phlorizin targets the apical SGLT1 transporter which is located in the apical membrane, mucosal application of selective SGLT1 inhibitor phlorizin (300 μM) significantly reduced MSG‐induced *I*_*sc*_, while selective SGLT2 inhibitor canagliflozin (10 μM) did not affect (Figure 5e,f), excluding the involvement of SGLT2. Taken together, these data suggest that SGLT1 also plays a certain role in MSG‐induced ileal *I*_*sc*_. SGLT1 likely plays a modulatory role via Na^+^ handling and competition with glucose rather than serving as a direct MSG transporter.

### 3.9 MSG induces Ca^2+^ signaling via SOCE/CRAC in IECs

**FIGURE 6:**
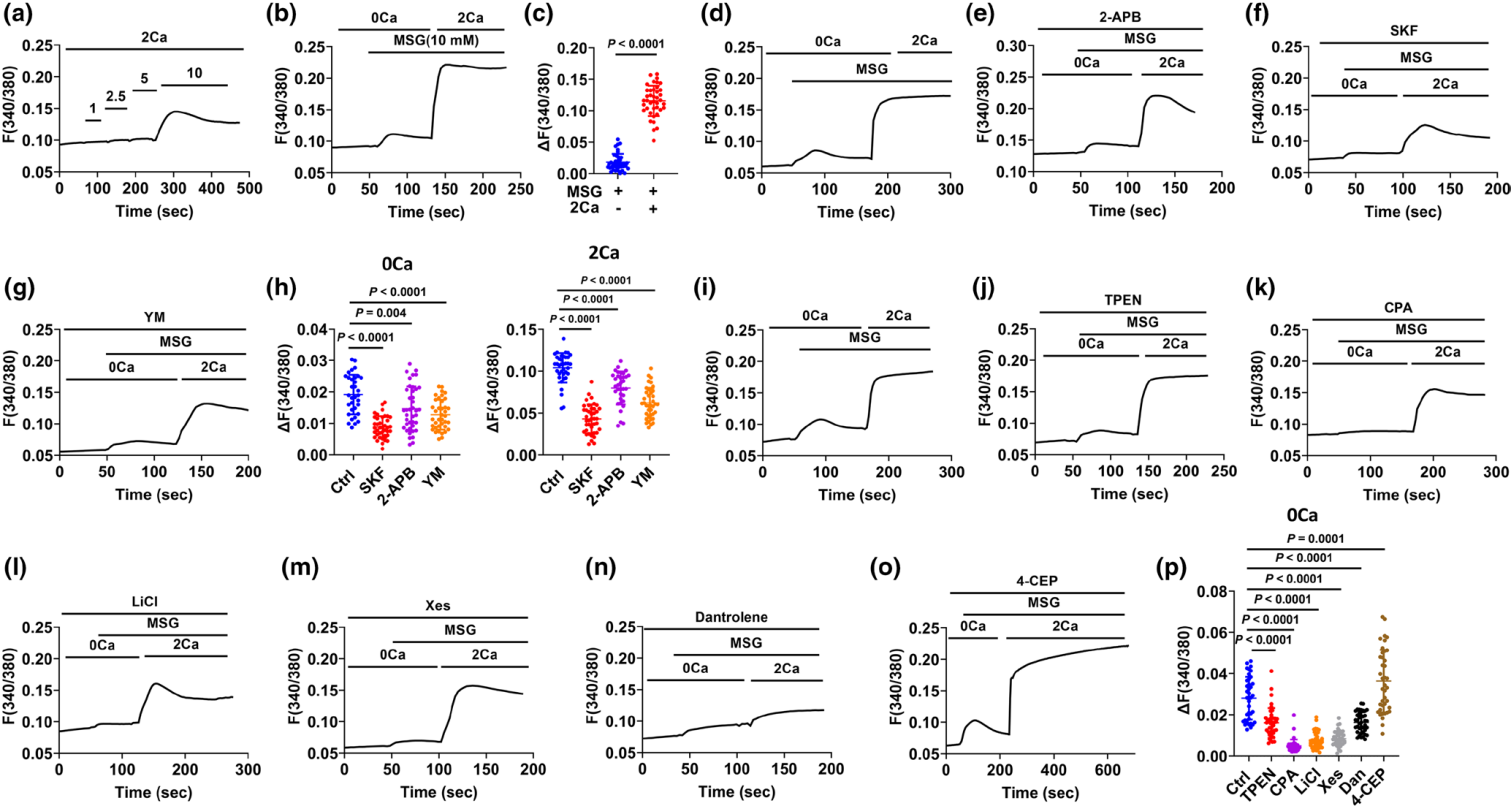
MSG induces Ca^2+^ signaling via SOCE/CRAC in IECs and the role of ER Ca^2+^ release via IP_3_R/RyR in it. (a) Summarizes the time course of Ca^2+^ signaling induced by different concentrations of MSG (1 mM, 2.5 mM, 5 mM, 10 mM). (b) Time courses of MSG (10 mM)‐induced Ca^2+^ signaling in the extracellular Ca^2+^ omission (0 Ca, left) or not (2 Ca, right) in IEC‐6 cells. (c) Summary data of the fluorescence ratios of Ca^2+^ signaling in IEC‐6 cells induced by MSG (10 mM) in the extracellular Ca^2+^ omission (0 Ca) or not (2 Ca) in IEC‐6 cells. (d) Time courses of MSG (10 mM)‐induced Ca^2+^ signaling in the extracellular Ca^2+^ omission (0 Ca, left) or not (2 Ca, right) in IEC‐6 cells. (e–g) Show the effect of 2‐aminoethoxydiphenyl borate (2‐APB, 20 μM), SKF‐96365 (SKF, 20 μM) and YM‐58483 (YM, 30 nM) on the time courses of MSG‐induced Ca^2+^ signaling in the absence or the presence of extracellular Ca^2+^. (h) Represents the summary data of the Δ Fura‐2 fluorescence ratios under 0 Ca and 2 Ca in d–g. (i) Time courses of MSG (10 mM)‐induced Ca^2+^ signaling in the extracellular Ca^2+^ omission (0 Ca, left) or not (2 Ca, right) in IEC‐6 cells. (j) Show the effect of TPEN (5 μM) on the time courses of MSG‐induced Ca^2+^ signaling in the absence or the presence of extracellular Ca^2+^. (k) Show the effect of CPA (10 μM) on the time courses of MSG‐induced Ca^2+^ signaling in the absence or the presence of extracellular Ca^2+^. (l) Show the effect of LiCl (3 mM) on the time courses of MSG‐induced Ca^2+^ signaling in the absence or the presence of extracellular Ca^2+^. (m) Show the effect of xestospongin C (Xes, 100 nM) on the time courses of MSG‐induced Ca^2+^ signaling in the absence or the presence of extracellular Ca^2+^. (n) Show the effect of dantrolene (Dan, 10 μM) on the time courses of MSG‐induced Ca^2+^ signaling in the absence or the presence of extracellular Ca^2+^. (o) Show the effect of 4‐CEP (100 μM) on the time courses of MSG‐induced Ca^2+^ signaling in the absence or the presence of extracellular Ca^2+^. (p) Represents the summary data of the Δ Fura‐2 fluorescence ratios under 0 Ca in i–o. *n* = 35–40 cells.

Since Ca^2+^ signaling plays a crucial role in regulating epithelial ion transport (Wan et al., 2023) and in maintaining cell functions of IECs (Petsakou et al., 2023), we thus examined whether MSG affected intracellular Ca^2+^ signaling in IEC‐6 cells using single cell Ca^2+^ imaging. Indeed, MSG (10 mM) significantly increased Ca^2+^ signaling (Figure 6a). To further determine the source of Ca^2+^ signaling, we first eliminated extracellular Ca^2+^. As shown in Figure 6b,c, in the absence of Ca^2+^ (0Ca), MSG (10 mM) still induced a small but significant Ca^2+^ signaling, and restoration of extracellular Ca^2+^ (2 mM Ca^2+^, 2Ca) induced a greater and sustained Ca^2+^ signaling. Therefore, MSG induces both intracellular Ca^2+^ release and extracellular Ca^2+^ influx in IEC‐6 cells.

Since we found earlier that SOCE/CRAC is critical for MSG‐induced intestinal *I*_*sc*_, we further examined its role in IECs. As shown in Figure 6d–h, two SOCE blockers, 2‐APB (20 μM) and SKF‐96365 (20 μM), as well as a selective CRAC blocker YM‐58483 (30 nM), significantly reduced MSG‐induced Ca^2+^ signaling in 0Ca and 2Ca solutions. These data confirm a critical role of SOCE/CRAC not only in MSG‐induced Ca^2+^ signaling in IECs but also in Ca^2+^‐mediated intestinal *I*_*sc*_ induced by MSG.

### 3.10 ER Ca^2+^ release via IP_3_R/RyR in MSG‐induced Ca^2+^ signaling

We further elucidated cellular mechanisms of MSG‐induced intracellular Ca^2+^ release in IEC‐6 cells. As shown in Figure 6i–k,p, MSG‐induced Ca^2+^ release (in 0Ca solutions) was significantly reduced by the ER Ca^2+^ chelator TPEN (5 μM) (Figure 6j,p) or the ER Ca^2+^ ATPase inhibitor CPA (10 μM) (Figure 6k,p). These data indicate that the ER Ca^2+^ store is crucial for MSG‐induced intracellular Ca^2+^ release.

Since IP_3_R and RyR play critical roles for ER Ca^2+^ release, we further explored their roles in MSG‐induced Ca^2+^ release. Both LiCl (3 mM) and xestospongin C (100 nM) significantly attenuated MSG‐induced intracellular Ca^2+^ release (Figure 6l,m,p). In addition, dantrolene (10 μM), a RyR inhibitor, also reduced MSG‐induced Ca^2+^ signaling (Figure 6n). Finally, we pretreated IEC‐6 cells with a selective RyR agonist, 4‐CEP (100 μM), which significantly potentiated MSG‐induced Ca^2+^ release (Figure 6o). Figure 6p summarizes the different effects of TPEN, CPA, LiCl, xestospongin C, dantrolene, and 4‐CEP on MSG‐induced Ca^2+^ release under 0Ca condition in IEC‐6 cells. These data demonstrate that ER Ca^2+^, IP_3_R and RyR are essential for MSG‐induced Ca^2+^ release, which in turn triggers SOCE/CRAC in IEC‐6 cells.

### 3.11 A coupling of NCX and SGLT1 in MSG‐induced Ca^2+^ and Na^+^ signaling in IECs

**FIGURE 7:**
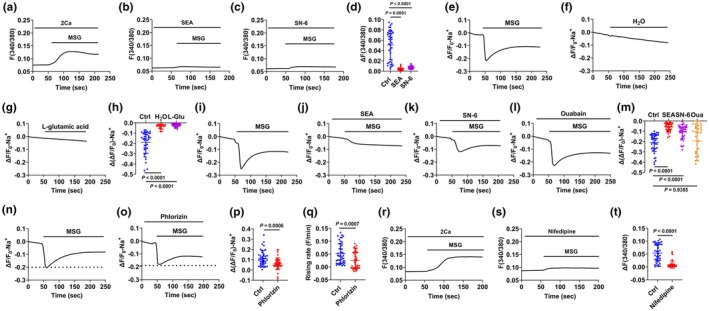
NCX, SGLT1 and VGCC in MSG‐induced Ca^2+^ and Na^+^ signaling in IECs. (a) Represents the time course of the changes in [Ca^2+^]_i_ induced by MSG (10 mM) in IEC‐6 cells. (b) Represents the time course of the changes in [Ca^2+^]_i_ induced by MSG after pretreatment with SEA0400 (SEA, 1 μM). (c) Represents the time course of the changes in [Ca^2+^]_i_ induced by MSG after pretreatment with SN‐6 (10 μM). (d) Represents the summary data of the Δ Fura‐2 fluorescence ratios in a–c. (e–g) Summary tracings of [Na^+^]_i_ time courses in response to MSG (10 mM), an equal volume of H_2_O and L‐glutamic acid (L‐glu, 10 mM) in IEC‐6 cells. (h) Summary data showing peak [Na^+^]_i_ signaling in e–g. (i–l) Summary tracings of [Na^+^]_i_ time courses in response to MSG (10 mM) after pretreatment with SEA0400 (SEA, 1 μM), SN‐6 (10 μM) and Ouabain (20 μM). (m) Summary data showing peak [Na^+^]_i_ signaling in i–l. (n, o) Summary tracings of [Na^+^]_i_ time courses in response to MSG (10 mM) after pretreatment with phlorizin (5 μM). (p) Summary data showing the peak recovered [Na^+^]i signaling in n–o. (q) Comparison of the rising rates of the recovered [Na^+^]i signaling in n–o. (r–t) The time course and summary data of the changes in [Ca^2+^]_i_ induced by MSG after pretreatment with nifedipine (1 μM). *n* = 35–40 cells.

Since it was previously demonstrated that NCX participates in regulating MSG‐induced ileal *I*_*sc*_, we further examined whether NCX is critical for MSG‐induced Ca^2+^ signaling in IECs. Two selective NCX inhibitors SEA0400 (1 μM) and SN‐6 (10 μM) significantly abolished MSG‐induced Ca^2+^ signaling in IEC‐6 cells (Figure 7a–d). The inhibition percentages of the above inhibitors on MSG‐induced Ca^2+^ signaling are listed in Table S2. To further validate the involvement of NCX in this process, we investigated Na^+^ dynamics upon MSG stimulation. Neither water nor L‐glutamic acid—the individual components of water‐soluble MSG—elicited similar changes in intracellular Na^+^ levels in the control study (Figure 7e–h). In contrast, MSG (10 mM) caused a significant decrease in intracellular Na^+^ levels, which was markedly attenuated by either SEA0400 (1 μM) or SN‐6 (10 μM) (Figure 7i–k). These results demonstrate not only that MSG induces a specific intracellular Na^+^ signaling in IECs, but also that MSG activates the Ca^2+^ influx mode of NCX, as evidenced by reduced intracellular Na^+^ levels coupled with enhanced Ca^2+^ signaling in IECs. In addition, considering that NKA transport is also accompanied by Na^+^ efflux, we used the selective inhibitor ouabain (20 μM) and found that the Na^+^ signaling did not differ significantly from that in the control group, fully demonstrating that MSG selectively targets NCX rather than NKA (Figure 7l,m). Interestingly, MSG induced a sharp decrease in intracellular Na^+^ levels, which was recovered quickly but not to the baseline (Figure 7e,i), suggesting Na^+^ may enter via SGLT1. To test this notion, we applied phlorizin (5 μM), a selective SGLT1 inhibitor, which significantly attenuated the recovery of MSG‐induced Na^+^ signaling by reducing its rising peak and rate (Figure 7n–q). These results demonstrate that MSG activates the Ca^2+^ influx mode of NCX, leading to a reduction in intracellular Na^+^ levels, while simultaneously activating SGLT1‐mediated Na^+^ influx to partially restore Na^+^ levels.

In addition, we demonstrated that VGCC was involved in MSG‐induced ileal *I*_*sc*_. Therefore, we further investigated the role of VGCC in MSG‐induced Ca^2+^ signaling in IECs. Indeed, the selective VGCC blocker nifedipine (1 μM) also abolished MSG‐induced Ca^2+^ signaling (Figure 7r–t), suggesting the involvement of VGCC in Ca^2+^‐mediated intestinal *I*_*sc*_ induced by MSG. The inhibition percentage of nifedipine on MSG‐induced Ca^2+^ signaling is shown in Table S2.

### 3.12 NKA, IK_Ca_ and SGLT1 in MSG‐induced Ca^2+^ signaling

**FIGURE 8:**
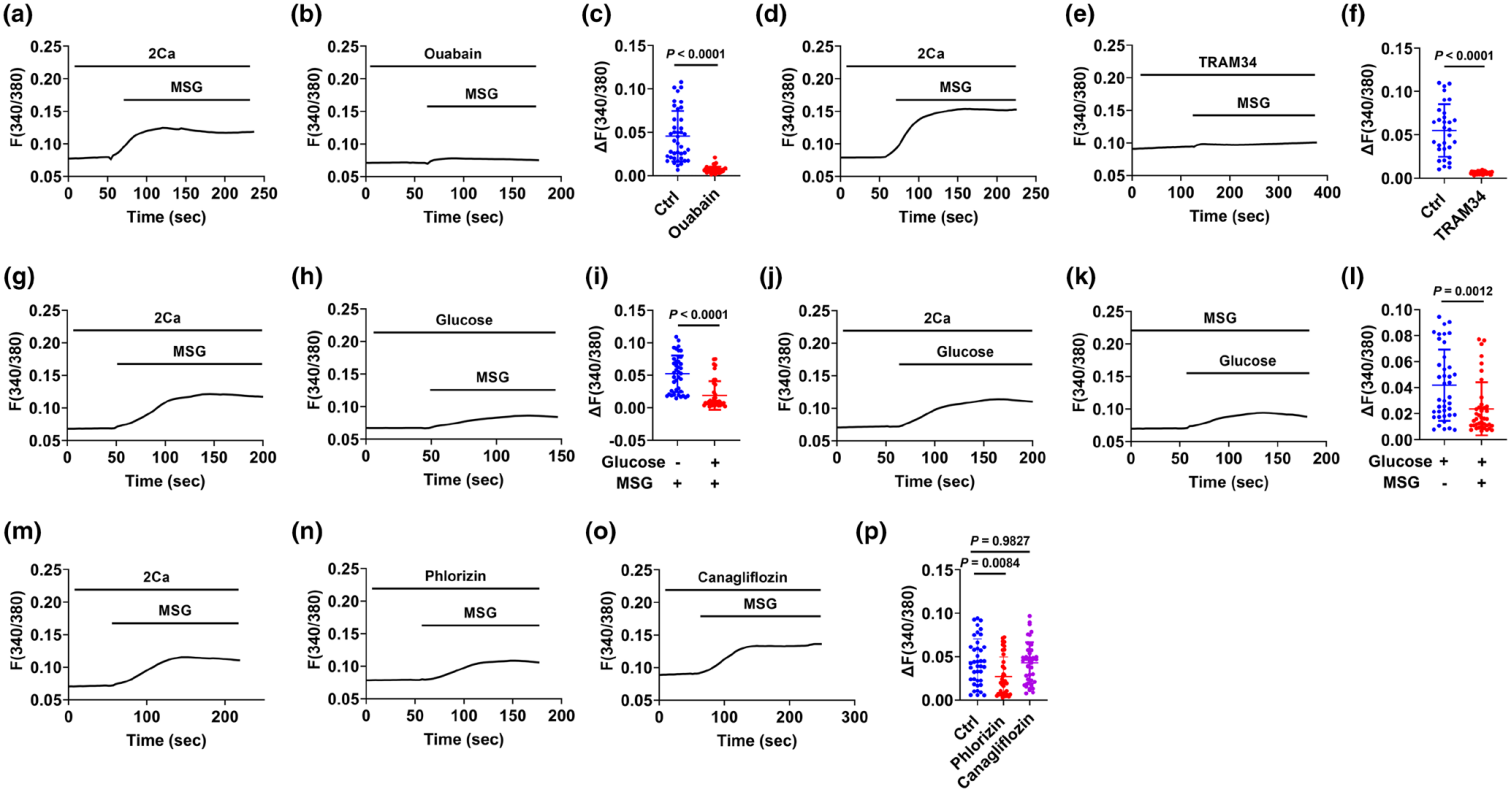
The role of NKA, IK_Ca_ and SGLT1 in MSG‐induced Ca^2+^ signaling. (a–c) The time course and summary data of the changes in [Ca^2+^]_i_ induced by 10 mM MSG after pretreatment with ouabain (20 μM). (d–f) The time course and summary data of the changes in [Ca^2+^]_i_ induced by 10 mM MSG after pretreatment with TRAM34 (1 μM). (g) The time course of the changes in [Ca^2+^]_i_ induced by MSG (10 mM). (h) Represents the time course of the changes in [Ca^2+^]_i_ induced by 10 mM MSG after pretreatment with glucose (3 mM). (i) Represents the summary data of the Δ Fura‐2 fluorescence ratios in g, h. (j) The time course of the changes in [Ca^2+^]_i_ induced by glucose (40 mM). (k) Represents the time course of the changes in [Ca^2+^]_i_ induced by 40 mM glucose after pretreatment with MSG (10 mM). (l) Represents the summary data of the Δ Fura‐2 fluorescence ratios in j, k. (m–p) The time courses and summary data of the changes in [Ca^2+^]_i_ induced by 10 mM MSG after pretreatment with phlorizin (10 μM) and canagliflozin (1 μM). *n* = 35–40 cells.

Since our previous studies showed that NKA and IK_Ca_ work in concert with SGLT1 to regulate MSG‐induced ileal *I*_*sc*_, we investigated whether MSG‐induced Ca^2+^ signaling is involved. As shown in Figure 8a–c, selective NKA inhibitor ouabain (20 μM) significantly reduced MSG‐induced Ca^2+^ signaling in IEC‐6 cells. Selective IK_Ca_ blocker TRAM34 (1 μM) also attenuated MSG‐induced Ca^2+^ signaling (Figure 8d–f). The inhibition percentage of the inhibitor on MSG‐induced Ca^2+^ signaling is shown in Table S2. Therefore, both NKA and IK_Ca_ play crucial roles in regulating MSG‐induced Ca^2+^ signaling.

Given that SGLT1 is crucial for MSG‐induced ileal *I*_*sc*_, we explored whether Ca^2+^ signaling is involved in this interaction. As shown in Figure 8g–i, MSG‐induced Ca^2+^ signaling in IEC‐6 cells was significantly attenuated by pretreatment with glucose (3 mM). Vice versa, glucose‐induced Ca^2+^ signaling was also significantly attenuated by pretreatment with MSG (10 mM) (Figure 8j–l), further confirming that MSG and glucose compete with each other for SGLT1. Furthermore, as shown in Figure 8m–p, MSG‐induced Ca^2+^ signaling was significantly attenuated by pretreatment with the selective SGLT1 inhibitor phlorizin (10 μM) but not with the selective SGLT2 inhibitor canagliflozin (1 μM). These results suggest SGLT1 likely plays a modulatory role via Na^+^ handling and competition with glucose.

### 3.13 MSG‐induced Ca^2+^‐dependent ion transports in IBS‐D

**FIGURE 9:**
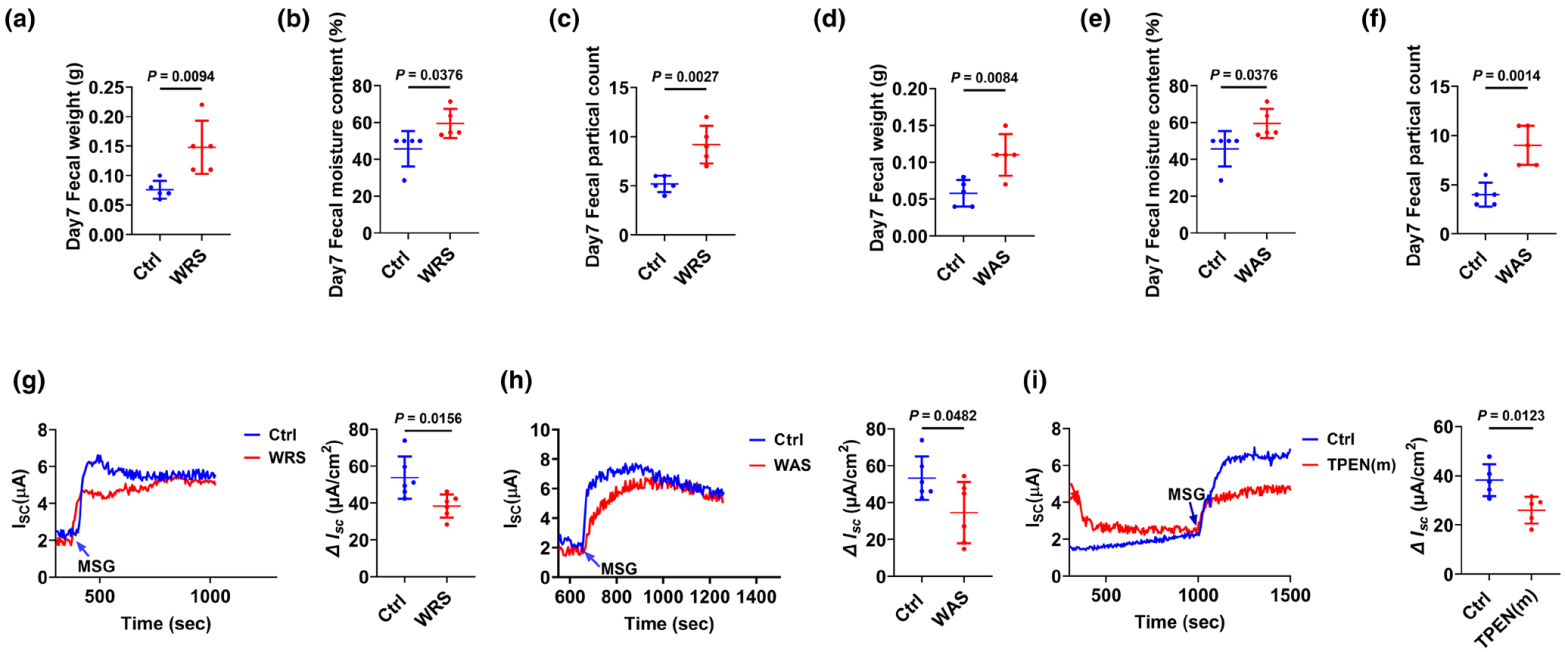
MSG‐induced Ca^2+^‐dependent ion transports in IBS‐D. (a–c) Summary data show the fecal weight, water content, and number of fecal particles in the control group and WRS group (*n* = 5). (d–f) Summary data show the fecal weight, water content, and number of fecal particles in the control group and WAS group (*n* = 5). (g) Representative time courses and summary data of MSG‐induced *I_sc_* in WRS (*n* = 6). (h) Representative time courses and summary data of MSG‐induced *I_sc_* in WAS (*n* = 6). (i) Representative time courses and summary data of MSG‐induced *I_sc_* when TPEN (1 mM, *n* = 6) was added to the mucosal side (m) in WRS.

Previous studies showed that MSG intake was associated with IBS (Holton et al., 2012), however, it is currently unclear whether MSG‐induced Ca^2+^‐dependent intestinal ion transports are changed in IBS‐diarrhea (IBS‐D). To explore whether MSG‐mediated ion transports are involved in IBS‐D, two classic mouse models, wrap restraint stress (WRS) and water‐avoidance stress (WAS), were applied (Xia et al., 2023; Xing et al., 2025). After restrained, the fecal weight, water content, and number of fecal particles in mice of the WRS group were significantly increased compared with the control group (Figure 9a–c). Similarly, these indicators were also considerably increased in WAS group mice (Figure 9d–f), indicating two IBS‐D mouse models were successfully established.

We further examined whether MSG‐mediated ion transports are involved in IBS‐D. In WRS mice, MSG‐induced ileal *I*_*sc*_ was lower than that in healthy mice (Figure 9g), and the same results were obtained in WAS mice (Figure 9h). Since our previous results proved that ER Ca^2+^ stores play a role in MSG‐induced ileal *I*_*sc*_ in healthy mice, we next explored whether this Ca^2+^ regulation is changed in IBS‐D. As shown in Figure 9i, TPEN (1 mM) on the mucosal side significantly attenuated MSG‐induced ileal *I*_*sc*_, suggesting the possible involvement of MSG‐induced Ca^2+^‐dependent ion transports in IBS‐D.

## 4 DISCUSSION

In the present study, using native mouse intestinal epithelium with preserved polarity and intestinal epithelial cell line IEC‐6 cells, we found that MSG: (1) induced Ca^2+^‐dependent intestinal ion transport via epithelial Na^+^ absorption and Cl^−^ secretion, as shown in Figures 1 and 2 activated the Ca^2+^ influx mode of NCX, Na^+^ absorption via SGLT1 and Cl^−^ secretion via CaCC and CFTR channels, as shown in Figures 2, 7, and 3 caused ER Ca^2+^ release via IP_3_R/RyR and subsequently triggered the SOCE/CRAC on the serosal side of the intestinal epithelium, as shown in Figures 3, 6, and 4 caused Ca^2+^ influx through VGCC and NCX, as shown in Figures 4, and 7, and 5 stimulated IK_Ca_ and NKA on the serosal side to jointly maintain intracellular Na^+^ and K^+^ for ion transport, as shown in Figures 5, 8, and 6 MSG‐induced Ca^2+^‐dependent ileal *I*_*sc*_ was changed in IBS‐D, as shown in Figure 9. Our study not only elucidates cellular mechanisms of intestinal effects of MSG in health, but also reveals a change in MSG‐induced ileal *I*_*sc*_ during IBS‐D.

As one of the most widely used food additives in the world, the biomedical effects of MSG and the mechanisms of action outside GI tract have been intensively investigated (Chakraborty, 2019). Although MSG first enters GI tract where it is digested and absorbed, surprisingly we know almost nothing about its actions on GI tract and intestinal epithelial ion transport, let alone the mechanisms of action. In the present study, as shown in Figure 1, 25 mM MSG was applied and induced a marked ileal *I*_*sc*_, verifying that MSG mediates ion transport across the intestinal epithelium. We selected a 25 mM concentration of MSG for the following reasons. First, this concentration elicits a sufficiently large *I*_*sc*_, enabling clear and quantitative assessment of MSG‐induced ion transport as well as subsequent modulation by pharmacological agents. Second, it lies substantially below the established threshold concentration, 1% MSG (approximately 53 mM), which previous study has demonstrated to impair epithelial barrier integrity and affect cellular progress including proliferation and differentiation (Zhao et al., 2025). The concentration used in our experiments is far lower than 53 mM, which neither significantly alters the osmotic pressure of intestinal tissues nor disrupts tissue physiological status. Moreover, it ensures the generation of a strong and stable current signal, guaranteeing the stability and reproducibility of experimental results. Meanwhile, we found that mucosal application of MSG evoked an upward *I*_*sc*_, whereas serosal MSG elicited a downward *I*_*sc*_. First, an increase in *I*_*sc*_ reflects cation absorption or anion secretion. We found that mucosal application of MSG promoted Na^+^ absorption, thereby inducing a marked upward *I*_*sc*_. In contrast, serosal addition of MSG elicited a downward *I*_*sc*_. We speculate that this phenomenon may be attributed to cation secretion or anion absorption triggered by serosal MSG exposure. For instance, MSG may activate basolateral potassium channels via Ca^2+^ signaling or other signaling pathways, allowing K^+^ to flow from the intracellular compartment toward the serosal side down its concentration gradient, which can be regarded as K^+^ secretion into the serosal compartment. This accounts for the reduction in *I*_*sc*_ following serosal MSG application. Nevertheless, oral MSG is primarily absorbed in the intestinal lumen; therefore, we still focused on the intestinal mucosal side as the main research object throughout this study. Meanwhile, we revealed for the first time that MSG increased an obvious intestinal *I*_*sc*_, which was significantly attenuated by removal of Cl^−^ and reduction in Na^+^. However, MSG‐induced *I*_*sc*_ in the distal ileum was much greater and faster than that induced in the duodenum and jejunum. This is consistent with glucose‐induced and glutamine‐induced *I*_*sc*_ although the exact reasons need further investigation (Cavin et al., 2020; Chu et al., 2022; Zhang et al., 2021). We presume that the distal ileum is probably the most sensitive and capable place for intestinal Na^+^‐coupled absorption because this place is the last checkpoint to prevent most nutrients from escaping to the large intestine. Indeed, the present study provides additional evidence to further support our previous notion.

As a well‐known second messenger, intracellular Ca^2+^ ([Ca^2+^]_i_) regulates several critical GI physiological functions, such as neural activity, hormone secretion, ion transport, motility, etc. However, the studies on MSG‐induced intestinal ion transport, especially the role of Ca^2+^ signaling in IECs, are currently lacking. The ileum is involved in the active Ca^2+^ transport when luminal Na^+^ coupled‐nutrients are high (Kellett, 2011), and it may play a more important role than the duodenum in adapting to hypocalcemia (Dhawan et al., 2017). Therefore, it is reasonable to speculate that MSG may cause Ca^2+^ and Na^+^ signaling interaction in IECs. In the present study, by applying native mouse intestine and IECs to investigate Ca^2+^ regulation of MSG‐induced ileal ion transport, we found an essential role of Ca^2+^ signaling for this regulation.

**FIGURE 10:**
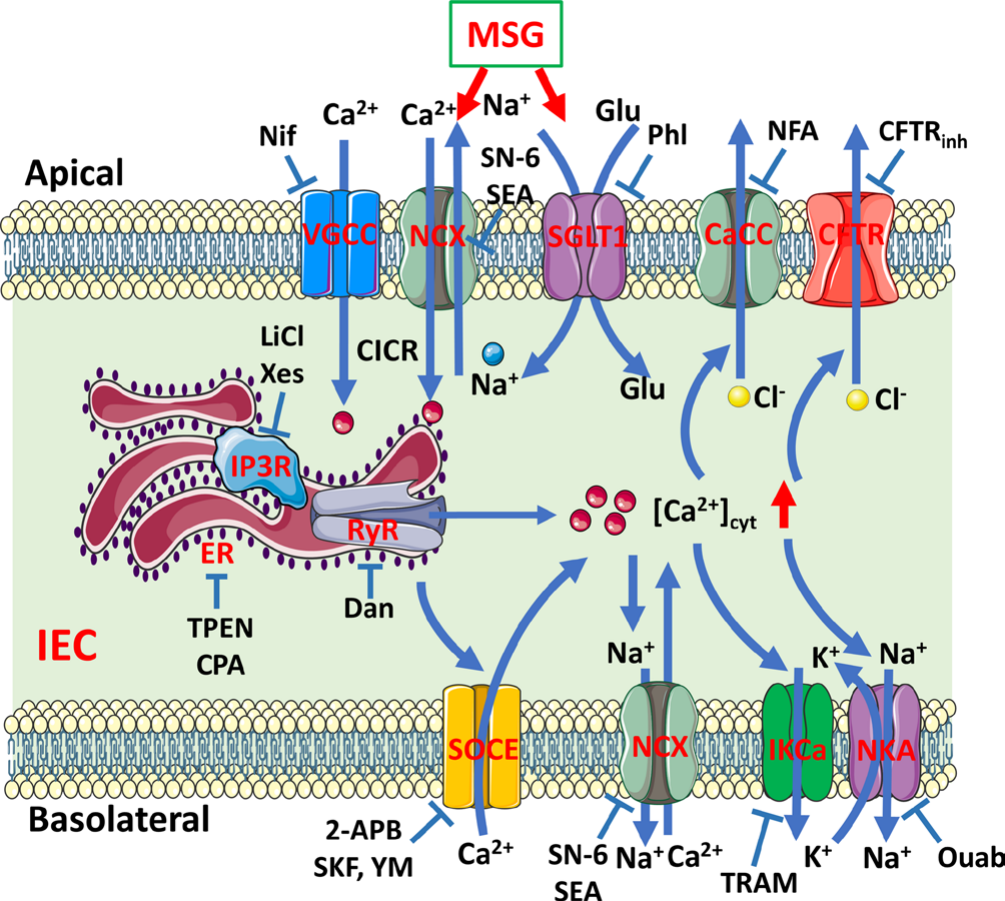
The schematic diagram depicts the mechanism of MSG‐mediated intestinal epithelial ion transport. MSG activates the Ca^2+^ influx mode of NCX and simultaneously activates SGLT1 to mediate Na^+^‐coupled glucose uptake. This synergistic increase in intracellular cations depolarizes the apical membrane, thereby triggering Ca^2+^ entry through VGCC in IECs. Ca^2+^ influx triggers CICR via RyR and IP_3_R on the ER, depleting ER Ca^2+^ stores and inducing SOCE/CRAC‐mediated basolateral Ca^2+^ influx. This further activates intermediate conductance IK_Ca_, NCX, and NKA to jointly maintain intracellular Na^+^ and K^+^ for ion transport. Ca^2+^ signaling stimulates Cl^−^ secretion through apical CaCC and CFTR channels, thereby regulating MSG‐induced epithelial ion transport. CaCC, Ca^2+^‐activated Cl^−^ channels; CFTR, cystic fibrosis transmembrane conductance regulator; CICR, calcium‐induced calcium release; CRAC, Ca^2+^ release‐activated Ca^2+^ channels; ER, endoplasmic reticulum; IK_Ca_, intermediate Ca^2+^‐activated K^+^ channel; IP_3_R, inositol 1,4,5‐triphosphate receptor; NCX, Na^+^/Ca^2+^ exchanger; NCX, Na^+^/Ca^2+^ exchanger; NKA, Na^+^/K^+^ ATPase; RyR, ryanodine receptor; SGLT1, sodium‐dependent glucose transporter 1; SOCE, store‐operated calcium entry; VGCC, voltage‐gated calcium channels.

As Figure 10 depicts, we demonstrate for the first time the cellular mechanisms of MSG on IECs: (1) [Ca^2+^]_i_ in IECs plays an essential role in the regulation of ileal MSG‐induced *I*_*sc*_; (2) MSG primarily activates the Ca^2+^ influx mode of NCX, and concurrently stimulates SGLT1, leading to elevated [Ca^2+^]_i_ and reduced intracellular Na^+^ levels, which likely depolarizes apical membrane to stimulate Ca^2+^ entry through VGCC in IECs; (3) Ca^2+^ induces Ca^2+^ release (CICR) via RyR and IP_3_R on the ER; (4) Ca^2+^ signaling stimulates serosal Ca^2+^ entry through SOCE/CRAC to further trigger IK_Ca_ channels, NCX and NKA; (5) Ca^2+^ signaling also stimulates Cl^−^ secretion via luminal CaCC and CFTR channels to regulate MSG‐induced epithelial ion transport. Therefore, our findings not only reveal an important role of [Ca^2+^]_i_ in the regulation of MSG‐induced intestinal ion transport but also provide new insights into cellular mechanisms of Ca^2+^‐mediated Cl^−^ secretion in the ileum.

There are two primary sources of Ca^2+^ in the cytoplasm in IECs: extracellular Ca^2+^ entry via plasma membrane and intracellular Ca^2+^ release from the ER. NCX is expressed in IECs to exist in two transport modes. In the Ca^2+^ efflux mode, 3 Na^+^ enter the cell while 1 Ca^2+^ is extruded. In the Ca^2+^ influx mode, 3 Na^+^ are extruded from the cell accompanied by the entry of 1 Ca^2+^ (Giladi et al., 2016). As shown in Figure 7, we demonstrate that MSG primarily activates the Ca^2+^ influx mode of NCX to simultaneously induce [Ca^2+^]_i_ rise but [Na^+^]_i_ reduction, which may activate SGLT1 in IECs. Since in the Ca^2+^ influx mode of NCX, 3 Na^+^ are extruded from cell accompanied by entry of 1 Ca^2+^ (Giladi et al., 2016), but only 2 Na^+^ influx into cell accompanied by entry of 1 glucose, MSG induced a sharp [Na^+^]_i_ decrease, which was recovered quickly but not to the baseline. These data suggest not only that Na^+^ may enter via SGLT1 but also that MSG may induce a coupling of the Ca^2+^ influx mode of NCX and SGLT1 in IECs. The selective SGLT1 inhibitor phlorizin significantly attenuated the recovery of MSG‐induced [Na^+^]_i_, which provides a solid evidence for MSG‐induced coupling of NCX and SGLT1. Therefore, this is the first time to demonstrate a functional coupling of NCX and SGLT1 in IECs.

Meanwhile, the VGCC has been reported to be expressed on the luminal side of small intestinal epithelium in mice (Beggs et al., 2019; Reyes‐Fernandez & Fleet, 2015). As shown in Figures 4 and 7, we reveal that Ca^2+^ entry through luminal VGCC plays a role in MSG transport, consistent with our previous reports on jejunal glucose and glutamine absorption (Chu et al., 2022; Zhang et al., 2021). MSG activates the Ca^2+^ influx mode of NCX and simultaneously activates SGLT1 to mediate Na^+^‐coupled glucose uptake. This synergistic increase in intracellular cations may depolarize the apical membrane, thereby further triggering Ca^2+^ entry through VGCC in IECs.

An increase in intracellular Ca^2+^ can further activate the inositol 1,4,5‐trisphosphate receptor (IP_3_R) and ryanodine receptor (RyR) on the endoplasmic reticulum (ER), thereby inducing the mechanism of Ca^2+^‐induced Ca^2+^ release (CICR) (Rahman, 2012; Zhang et al., 2021). This mechanism was corroborated by our findings in the ileum: ileal MSG‐induced *I*_*sc*_ was significantly inhibited by depleting the ER Ca^2+^ store with CPA or TPEN, suppressing IP_3_ production, or specifically blocking IP_3_R and RyR, as shown in Figures 2 and 3. Depletion of the ER Ca^2+^ store triggers extracellular Ca^2+^ influx via CRAC channels, a process known as SOCE, as shown in Figure 6. This SOCE process plays a critical role in regulating various Ca^2+^‐dependent physiological functions in IECs, such as in jejunal glucose absorption (Zhang et al., 2021) and ileal glutamine transport (Chu et al., 2022). Furthermore, multiple ion channels and transporters are functionally expressed on the serosal side of the small intestinal epithelium (Cui et al., 2021; Dong et al., 2005; Manneck et al., 2021; Tsuchiya et al., 2014). Notably, although the IK_Ca_ and NKA on the serosal side are conventionally recognized as fundamental components that provide the driving force for epithelial ion transport, the present study found that they act in concert to mediate the MSG‐induced *I*_*sc*_ in the ileum, as shown in Figures 5 and 8. In conclusion, several ion channels and transporters located on the serosal side of the ileum likely work together synergistically to regulate the Ca^2+^‐dependent MSG‐induced *I*_*sc*_.

While the cAMP/PKA and cGMP/PKG axes are well‐established drivers of Cl^−^ secretion in IECs, Ca^2+^ constitutes another critical signaling node governing this process (Flemström & Isenberg, 2001; Jung & Lee, 2014). However, the precise Ca^2+^‐dependent machinery driving anion secretion in native intestinal tissue has remained elusive. Current understanding indicates that Ca^2+^ regulates anion efflux not only via CaCC, but also through the modulation of CFTR. Specifically, Ca^2+^ can promote CFTR activity via: activation of adenylyl cyclase isoforms (resulting in PKA‐mediated CFTR phosphorylation), stimulation of the Pyk2/Src pathway which both directly phosphorylates CFTR and inhibits protein phosphatase type 2A‐mediated dephosphorylation in airway cells (Bagur & Hajnóczky, 2017), and upregulation of PI_3_K/Akt‐dependent CFTR phosphorylation in the intestine (Yang et al., 2018). In the present study, we demonstrate that MSG may stimulate intestinal Ca^2+^‐dependent Cl^−^ secretion via activation of CaCC and CFTR channels, as shown in Figures 1 and 2. This raises one question of why inhibitors of CaCC and CFTR suppress MSG‐induced *I*_*sc*_ on the serosal side, even though these channels are expressed on the intestinal mucosal side. The possible explanations are as follows: (1) The intestinal mucosal surface is covered by a continuous mucus barrier that selectively restricts the transmucosal penetration of substances (Atuma et al., 2001). (2) Functional activation of CFTR‐mediated Cl^−^ secretion depends on the elevation of intracellular second messenger cAMP, and the core molecules involved in its regulation (adenylyl cyclase, upstream activators of PKA) are mainly localized on the serosal side (Fiedorczuk et al., 2024; Ostrom et al., 2022). Similarly, activation of CaCC‐mediated Cl^−^ secretion relies on elevated intracellular Ca^2+^, and the sustained elevation of intracellular Ca^2+^ primarily depends on the serosal SOCE mechanism. When the ER Ca^2+^ store is depleted, the Orai1 channel (a core component of SOCE) on the serosal side opens to mediate extracellular Ca^2+^ influx (Wan et al., 2023), and SOCE components such as Orai1 are also clearly localized on the serosal side of epithelial cells (Kodakandla et al., 2023). These findings may explain the observed inhibitory effect of CaCC and CFTR inhibitors on MSG‐induced *I*_*sc*_ on the serosal side.

Irritable bowel syndrome (IBS) is one of the most prevalent chronic functional gastrointestinal (GI) disorders in humans, characterized primarily by abdominal pain, bloating, and alterations in bowel habits without detectable evidence of organic lesions in the intestine (Altomare et al., 2021). Due to predominant symptoms, IBS is divided into four groups: predominant constipation (IBS‐C), predominant diarrhea (IBS‐D), mixed bowel habits (IBS‐M) and the predominant symptoms are unclassified (IBS‐U). The changes in intestinal ion transports are involved in IBS although the detailed pathogenesis is currently unclear and multiple factors may participate (Alammar & Stein, 2019; Fukudo & Kanazawa, 2011; Noemi et al., 2024). Previous studies showed that MSG intake was associated with IBS (Holton et al., 2012); however, it is currently unclear whether MSG‐induced Ca^2+^‐dependent intestinal ion transports are changed in IBS. In our study, after establishing two classic IBS‐D models, the wrap restraint stress (WRS) and the water‐avoidance stress (WAS), as shown in Figure 9, we found tha MSG‐induced ileal *I*_*sc*_ was significantly attenuated, indicating reduced Na^+^ absorption and Cl^−^ secretion. Furthermore, MSG‐induced ileal *I*_*sc*_ in IBS mice was significantly reduced by ER Ca^2+^ chelator TPEN, suggesting a Ca^2+^‐dependent mechanism. Therefore, a reduction in MSG‐induced Ca^2+^‐dependent intestinal ion transports is likely involved in the process of IBS‐D; however, further study is needed to elucidate the underlying mechanisms.

In conclusion, we have provided novel insights into the intestinal effects of MSG and revealed for the first time that it mediates Ca^2+^‐dependent intestinal Cl^−^ secretion via activation of multiple epithelial ion channels and transporters, particularly NCX and SGLT1 in IECs. MSG‐induced intestinal ion transports are likely changed during IBS‐D. We not only elucidate cellular mechanisms of MSG‐induced Ca^2+^‐dependent epithelial ion transports but also uncover their possible involvements in IBS‐D. These findings provide novel insights into the intestinal effects of MSG in health and in IBS‐D. These new findings offer important experimental evidence for the potential impacts of daily intake of MSG on human GI health and water‐electrolyte balance. In addition, since MSG has central neural effects (Kim et al., 2025; Ureña‐Guerrero et al., 2003) and is likely associated with the progression of some GI diseases, such as irritable bowel syndrome and inflammatory bowel disease (Brant et al., 2023), our data also imply the possible involvements of MSG‐induced intestinal ion transport in these common and critical GI diseases as evidenced by a recent study (Zhao et al., 2025), which needs urgent further investigation.

## AUTHOR CONTRIBUTIONS

**Yunxian Wang:** Data curation; formal analysis; investigation; software; validation. **Fenglan Chu:** Data curation. **Yanhua Gong:** Data curation. **Ruihong Guo:** Methodology; supervision. **Hui Dong:** Conceptualization; funding acquisition; methodology; project administration; supervision.

## FUNDING INFORMATION

MOST | National Natural Science Foundation of China (NSFC): Hui Dong, No. 82570647 and 82273115 to H.D.

## CONFLICT OF INTEREST STATEMENT

The authors declare no conflicts of interest.

## ETHICS STATEMENT

The animal use protocol has been reviewed and approved by the Ethics Committee of the Medical College of Qingdao University (QDU‐AEC‐2025102) and was conducted in compliance with its guidelines.

## CONSENT

The authors declare consent for publication.

## Supporting information


**Table S1:** Summary of reagents.

**Table S2:** Inhibition percentage of different inhibitors on *I*_*sc*_ and [Ca^2+^]_i_.

## Data Availability

The data that support the findings of this study are available from the corresponding author upon reasonable request.
