# Supplementary material for: Monosodium glutamate‐mediated Ca2+‐dependent intestinal epithelial ion transports in health and IBS‐D in male mice
Source: Physiol Rep. 2026 Jun 12;14(11):e70975. doi: 10.14814/phy2.70975 (PMC13261090; doi:10.14814/phy2.70975)
Supplement: Supplementary file 1 — Table S1: Summary of reagents. [file PHY2-14-e70975-s002.docx]

**Supplementary Table 1.** Summary of reagents

| **Reagents** | **Source** | **Cat** |
| --- | --- | --- |
| Nifedipine | Aladdin | N123275 |
| Magnesium D-gluconate hydrate | Aladdin | M112987 |
| Monosodium glutamate (MSG) | Sigma | 49621 |
| Cyclopiazonic acid (CPA) | Sigma | T15027 |
| 4-chloro-3-ethylphenol (4-CEP) | Sigma | 279552 |
| L-glutamic acid | MedChemExpress | HY-14608 |
| TPEN | MedChemExpress | HY-100202 |
| Dantrolene | MedChemExpress | [HY-12542](https://www.medchemexpress.cn/dantrolene.html) |
| SN-6 | MedChemExpress | HY-107658 |
| Xestospongin C | MedChemExpress | HY-103312 |
| Ouabain | MedChemExpress | HY-B0542 |
| 2-Aminoethyl diphenylborinate (2-APB) | MedChemExpress | HY-W009724 |
| SKF-96365 | MedChemExpress | HY-125942 |
| YM-58483 | MedChemExpress | HY-100831 |
| Niflumic acid (NFA) | MedChemExpress | HY-B0493 |
| CFTR_inh_-172 | MedChemExpress | HY-16671 |
| LiCl | Macklin | L812571 |
| TRAM34 | Macklin | T849233 |
| SEA0400 | Macklin | S872663 |
| Phlorizin | Macklin | P816784 |
| Canagliflozin | Macklin | C857179 |
| KCl | Sangon | A501159 |
| D-(+)-Glucose | Sangon | A501991 |
| NaCl | Sangon | A610476 |
| CaCl_2_ | Sangon | A501330 |
| Sodium D-gluconate | Sangon | [A500852](javascript:voild(0)) |
| Sodium bicarbonate | Sangon | A500873 |
| Potassium phosphate monobasic | Sangon | A501211 |
| **Reagents** | **Source** | **Cat** |
| D-Mannitol | Sangon | A600335 |
| Potassium-D-gluconate | Sangon | A507810 |
| Calcium gluconate monohydrate | Sangon | A500254 |
| Potassium phosphate dibasic anhydrous | Sangon | A501212 |
| Agarose H | Sangon | A500016 |
