# Supplementary material for: Monosodium glutamate‐mediated Ca2+‐dependent intestinal epithelial ion transports in health and IBS‐D in male mice
Source: Physiol Rep. 2026 Jun 12;14(11):e70975. doi: 10.14814/phy2.70975 (PMC13261090; doi:10.14814/phy2.70975)
Supplement: Supplementary file 2 — Table S2: Inhibition percentage of different inhibitors on I sc and [Ca2+]i. [file PHY2-14-e70975-s001.docx]

**Supplementary Table 2. Inhibition percentage of different inhibitors on *I_sc_* and [Ca^2+^]_i_**

| **Inhibitor** | **Target** | **Inhibition rate of *I_sc_* (%)** | **Inhibition rate of ([Ca^2+^]_i_) (%)** |
| --- | --- | --- | --- |
| SN-6 | NCX | \| 39.61935 \| \| --- \| | \| 85.25105 \| \| --- \| |
| SEA | NCX | \| 34.87654 \| \| --- \| | \| 92.03555 \| \| --- \| |
| Nifedipine | VGCC | \| 59.09001 \| \| --- \| | \| 83.11423 \| \| --- \| |
| Ouabain | NKA | \| 47.17139 \| \| --- \| | \| 85.07793 \| \| --- \| |
| TRAM34 | IKCa | \| 28.07406 \| \| --- \| | \| 88.41449 \| \| --- \| |
